# Supplementary material for: Lessons learnt about implementing LEGO based therapy (Play Brick Therapy) based on fidelity data and experience from a large school-based randomised controlled trial
Source: PLoS One. 2026 Feb 4;21(2):e0336952. doi: 10.1371/journal.pone.0336952 (PMC12872000; doi:10.1371/journal.pone.0336952)
Supplement: S1 Fig — (DOCX) [file pone.0336952.s001.docx]

S1 Fig. Pre-liminary logic model for Play Brick Therapy based on the trial conduct and delivery.
