# Supplementary material for: Lessons learnt about implementing LEGO based therapy (Play Brick Therapy) based on fidelity data and experience from a large school-based randomised controlled trial
Source: PLoS One. 2026 Feb 4;21(2):e0336952. doi: 10.1371/journal.pone.0336952 (PMC12872000; doi:10.1371/journal.pone.0336952)
Supplement: Attachment — Submitted filename: PLOS review_20241106.pdf [file pone.0336952.s002.pdf]

Reviewer’s comments

Lessons learnt about implementing LEGO® based therapy (Play Brick Therapy) from a large school-based randomised controlled trial.

[Title and Abstract]

This article details the implementation fidelity of LEGO® based therapy, which is implemented through school-based randomised controlled trials. This topic is important as it highlights the importance of process evaluation, which measures to what extent the intervention is implemented on the ground and offers insights for further improvement. In particular, stemming from the original impact evaluation study by (Wright et al., 2023), the current article focuses on the results from a small portion of the sample schools (9%) that participated in the independent fidelity assessment. However, the title and abstract have not addressed the scope and purpose of the current study and possibly misleading readers to expect a complete result of the impact evaluation. For example:

- The title and introduction need to clearly mention the implementation ‘fidelity’ as a core of the current study;
- RCT should not be highlighted, as the data used in this study only covers sub-groups of the treatment group;
- Methods need to be applied to the analytical sample of this study (22 schools participated in the independent fidelity assessment) rather than the entire sample from the original study. What method is used for the data analysis in the study, e.g., descriptive statistics?

[Introduction/ The Research Study]

- It may benefit from the detailed summary of Wright et al., (2023a) as a motivation for the current study.
- What is the age group of ‘young’ people from primary and secondary schools? Did the play-based approach adapt to age-appropriate practices to cover a wide range of age groups?
- (Trial Design and Delivery section) The programme was delivered within 12 weeks, but was there any reason to administer midline/endline assessments in 20 and 52 weeks after a few months from the programme implementation? The time gap between the intervention and assessment could explain the diminishing impact revealed in Wright et al. (2023a).
- (Table 1) The authors need to present this with the number of schools and students, as well as the distribution of treatment and control groups. However, it should be clear that this is not the sample of the following analysis in the results section.
- The purpose of the research and research questions need to be described explicitly. The first results on LEGO-based therapy delivery are descriptive statistics about the facilitator’s profile, previous knowledge or experience of autism/other interventions. The

second results cover descriptive statistics of independent fidelity assessment through the rating consistency between facilitators and independent facilitators.

- (pg. 10 in PDF review file) The authors said, "*Further details of the fidelity results are reported in Wright et al 2023b but does not include the analysis or discussion in this paper.*" Please give more details about what you included and what you did not include in this paper.
- (pg. 13 in PDF review file)
  - Why was self-reported fidelity analysed by sessions, not by facilitators?
  - In total, 22 schools were included in the independent fidelity assessment – how were they selected? Had this group of schools any representativeness?
  - You need to use headings and subheadings properly. For example, the facilitation of social interaction, structural quality, and adherence to the programmes should be under the analysis of trends.
  - Also, it is not clear why this showed 'trends'—was there any measurement of changes over time?
- (Table 6) "*Fidelity rating by item and points of disagreements between facilitator and independent ratings are reported in Table 6.*"
  - The authors need to provide a description and interpretation of Table 6 in the results section.
  - All items have 63 responses (but there are 62 responses in some rows without any missing variable – e.g., Did you highlight some aspect of social interaction and prompt children to problem solve or discuss it with each other?). How many facilitators and independent evaluators were included in this survey? It is unclear who are the independent trained researchers.

[Discussion]

- The overall article is very descriptive without probing the implementation fidelity more rigorously. For example, it would be more meaningful to see the relationship between the facilitator's profile/experience and their score in the independent fidelity assessment (agreement/disagreement).
- The discussion section is rich in locating the findings in the literature. However, there are some redundant parts – e.g., three hours of training is too short. It could be more structured by implementation barriers that emerged from the study: knowledge or experience issues, time (or resource) constraints for training, etc.
- Although there is a separate limitations section, the authors listed limitations previously in the discussion section.
- Implications could be converged to conclusions.
